# Congenital Midline Cervical Cleft

**Published:** 2013-01-01

**Authors:** Bilal Mirza

**Affiliations:** Department of Pediatric Surgery, The Children's Hospital and the Institute of Child Health Lahore, Pakistan

A 3-year-old boy presented with a midline skin defect in the center of the neck. On examination, a 6 cm long and about 1.5 cm wide lesion extending from hyoid bone to the suprasternal notch was noted. There was a nipple like skin hood at the cranial end of the defect with a sinus at the caudal part (Fig. 1). 

**Figure:**
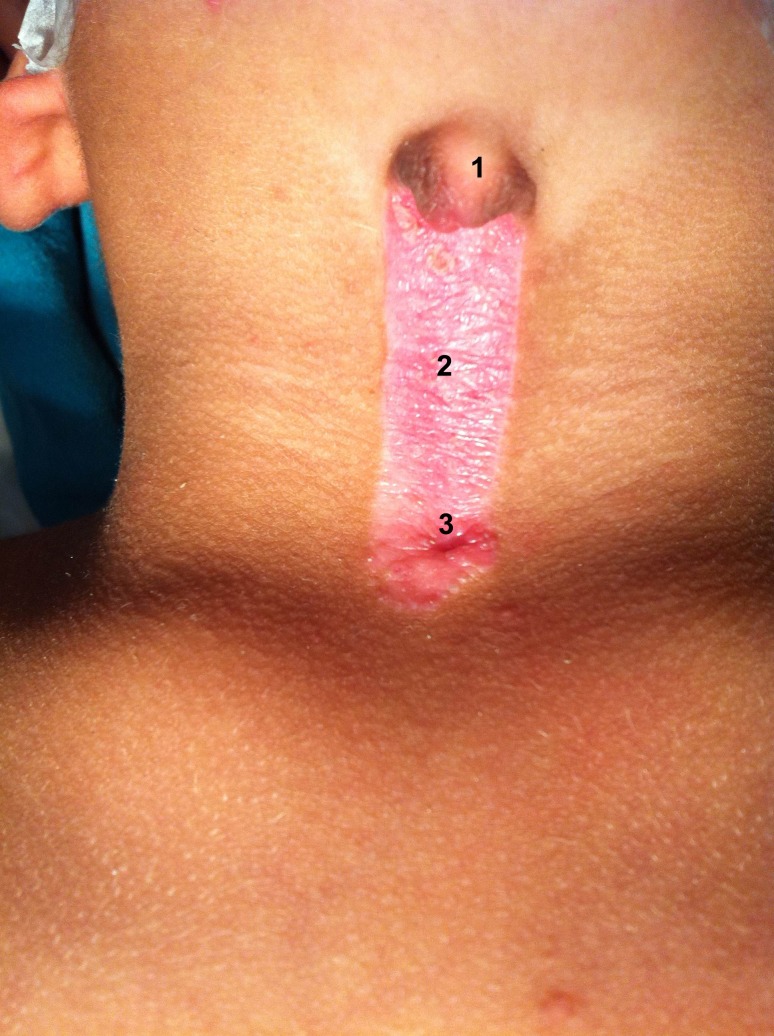
Figure 1: Congenital midline cervical cleft showing nipple like hood (1), cleft (2), and sinus (3).

On palpation, sub-mental bony spur was felt. The defect was associated with limitation of neck extension. Surgery was offered. At operation the extent of the sinus was about 4-6mm. The skin defect along with its cranial and caudal lesions was excised. The surgical wound was closed with multiple z-plasties (Fig. 2). The postoperative recovery was uneventful. Histopathology of the specimen showed skin histology in the upper hood like part. The cleft portion was devoid of skin appendages, and the sinus tract was blind and lined by pseudostratified ciliated columnar epithelium. At follow up the neck extension has greatly improved. The parents were satisfied with the cosmetic outcome.

**Figure:**
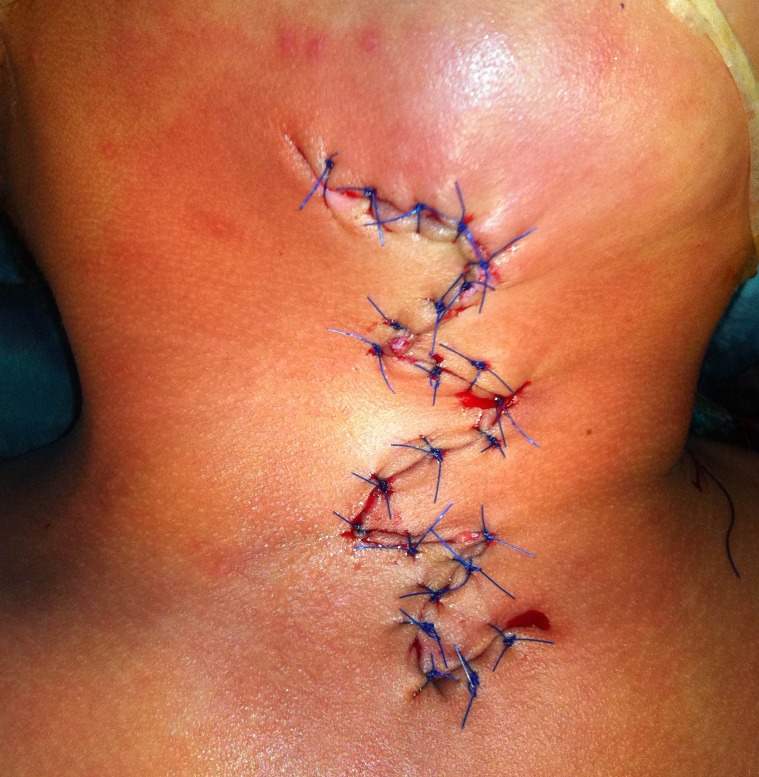
Figure 2: Wound closure after excision of the defect.

## DISCUSSION

Congenital midline cervical cleft (CMCC) is a rare anomaly and less than 100 cases have been reported to date [1]. The prevalence of CMCC in all cases of thyroglossal cyst and brachial cleft sinuses is 1.7% and it is considered a variant of Tessier 30 craniofacial cleft [1, 2]. It presents at birth as a visible defect in cervical region in the midline. CMCC consists of three parts, a nipple like skin hood at cranial end followed by a middle area of skin cleft with a small blind ending sinus caudally. Embryologically, most of the authors believe it to be the outcome of failure of branchial arches I and II, to fuse in the midline. The main indications of surgery are cosmetic, prevention of neck contracture, release of neck contracture in case of delayed presentation, and to some extent psychological effect on the patient. If left untreated for a longer period, the lesion would result in severe neck contracture. My patient presented at the age of 3 year with considerable limitation of extension of the neck. These defects can be excised elliptically with closure of the defect with and without Z-plasty. In case of small defect one Z-plasty can be sufficient, however in large defects, as in index case, multiple z plasties have to be performed. Some authors advocate use of skin flaps to cover the defect [1-5].

## Footnotes

**Source of Support:** Nil

**Conflict of Interest:** None declared

## References

[1] ( 2012). Sinopidis X, Kourea HP, Panagidis A, Alexopoulos V, Tzifas S, Dimitriou G, et al. Congenital midline cervical cleft: diagnosis, pathologic findings, and early stage treatment. Case Rep Pediatr.

[2] Warden C, Millar A J W (2010). A rare congenital midline cervical cleft. SAJS.

[3] Spencer Cochrane C, DeFatta R J, Brenski A C (2006). Congenital midline cervical cleft: a practical approach to z-plasty closure. Int J Pediatr Otorhinolaryngol.

[4] Mlynarek A, Hagr A, Tewfik T L, Nguyen V H (2003). Congenital mid-line cervical cleft: case report and review of literature.. Int J Pediatr Otorhinolaryngol.

[5] Eastlack J P, Howard R M, Frieden I J (2000). Congenital midline cer-vical cleft: case report and review of the English language literature. Ped Derm.

